# Thermographic Study of the Orofacial Structures Involved in Clarinetists Musical Performance

**DOI:** 10.3390/dj6040062

**Published:** 2018-11-01

**Authors:** Ana Barros, Joaquim Mendes, André Moreira, Ricardo Vardasca, Miguel Pais Clemente, Afonso Pinhão Ferreira

**Affiliations:** 1 Faculty of Dental Medicine, University of Porto, 4200-393 Porto, Portugal; anapbbarbosa@gmail.com (A.B.); andre.luis.sa.moreira@gmail.com (A.M.); miguelpaisclemente@hotmail.com (M.P.C.); 2 INEGI-LAETA, Faculty of Engineering, University of Porto, 4200-465 Porto, Portugal; ricardo.vardasca@fe.up.pt; 3 Department of Orthodontics, Faculty of Dental Medicine; University of Porto, 4200-393 Porto, Portugal; aferreira@fmd.up.pt

**Keywords:** thermography, wind instrument, clarinet, musculoskeletal apparatus

## Abstract

Introduction: Wind instrumentalists like clarinetists, may present a muscular hyperactivity on certain groups of the cranio-cervico-mandibular complex, due to their musical activity. Therefore, the use of infrared thermography can be used to assess and characterize the orofacial structures involved in clarinet performance. Aim: The objective of this study was to analyze and record the thermal patterns using the thermographic camera Flir^®^ E60sc to evaluate anatomically and physiologically certain orofacial structures of the cranio-cervical-mandibular complex, such as the masticatory muscles and the region of the temporomandibular joint. Methodology: A sample of 30 clarinetists completed an individual questionnaire composed of two components (musical and clinical history of the participant), and were subjected to a clinical examination. Four thermographic images were taken of the cranio-cervical-mandibular complex at a rest position with frontal, right lateral, left lateral and anterior dentoalveolar components views. Each musician performed a piece of music for an uninterrupted period of 10 min. New thermographic images were captured with the same incidences, after the performance, respecting the same protocol. Results: There were statistically significant differences in the areas corresponding to the left temporal muscle, the orbicularis muscle (labial component), the left and right perioral teguments, as well as in the upper central incisors. There was also statistical evidence regarding the initial and final temperature asymmetries regarding temporal muscle and orbicular muscles (labial and marginal components). Conclusion: Infrared thermography has been shown to be an effective complementary diagnostic tool in the monitorization of the cranio-cervical-mandibular complex of clarinetists.

## 1. Introduction

The wind instrumentalists are part of a population that aims to achieve standards of excellence during their musical performance. These professionals are submitted to certain physical requirements, given the significant number of hours of practice and consequent adopted postures. The cognitive component is strongly developed by the execution of musical pieces and memorization of possible excerpts, which happens with more notoriety in solo musicians.

These instruments can be classified, according to the material of the instruments, as woodwind and brass [1]. Among these we can sub-classify the woodwind instrumentalists in single reed instrumentalists, like the clarinet and the saxophone, and double reed instrumentalists, like the bassoon and the oboe. In the group of metals there are instruments like the trumpet, the trombone, the tuba, and the horn.

In this research, the sample was constituted by clarinetists, being this the reason why more attention was paid to the description of the relationships of orofacial structures involved in musical practice of this instrument.

The position adopted by the clarinetists mouthpiece inside the oral cavity is in relation with the upper central incisors, the upper lip, the lower lip, and the tongue [2,3] The lower lip rests on top of the lower incisor edges, functioning as a sort of “cushion” between the mouthpiece superiorly stabilized by the two central incisors. The relation of the mouthpiece regarding these orofacial structures can eventually induce changes in the dental position due to the practice of clarinet [3], the appearance of orofacial pain in the surrounding structures [4], as well as an increase in the functional activity of the temporomandibular joint and the different muscle groups constituting the cranio-cervical-mandibular complex (CCMC) [5,6,7,8,9,10]. The occurrence of this muscular hyperactivity during the musical practice of the clarinetist, arouses interest in the analysis of the perioral structures, since there is a significant effort in the musculature involved [11,12,13].

However, due to the competitive nature of the socio-professional environment of these wind instrumentalists, most of them tend to avoid medical care, even when the consequence of excessive performance can generate the presence of pain [6]. The fear of impairing the evolution of instrumental practice overrides the need for medical evaluation. In fact, the continuous musical activity, together with short rest periods, discourages the search for medical help. Nevertheless, some musicians with a greater sense of concern seek for consultation when they are not overwhelmed at work [7,8,9].

In recent years, there have been links between art and science, namely with the presentation of studies of the implications of the musical instrumentalist activity on oral health [14,15,16,17,18,19]. This connection is feasible, since dental medicine has tools capable of diagnosing and treating orofacial problems, with etiology in musical practice [2].

One of the analytical methods that can be used is infrared medical thermography, which has been applied in different specialties [14]. The musculoskeletal system has been one of the target areas of greatest interest and studied by the scientific community [15]. The application of infrared thermography in dental medicine, as a complementary method of diagnose and therapeutic has been shown to be a valid tool for the characterization and monitoring of orofacial structures [15]. In performing arts medicine, this technique of infrared has also been used in the analysis study of the performance of wind instrumentalist [2,20]. This technology presents advantages for the study of the orofacial structures involved in clarinetists musical performance, since it is portable, fast, non-contact, and non-invasive, and presents no risk as it uses non-ionized radiation [21,22,23].

## 2. Methodology

The sample consisted of 30 clarinetists volunteers for this study, with a degree in the area of Music, more specifically clarinet, or students in recognized and prestigious higher education institutions such as, Escola Superior de Música e Artes do Espectáculo, ESMAE–Porto and the Department of Communication and Art of the University of Aveiro (DeCA). In order to be able to complete our sample, some clarinetists were also included who performed functions in institutions with programs of high artistic level, such as the Portuguese Symphonic Band (BSP) and the Military Band of the Oporto Army.

For this study, inclusion criteria was defined as professional clarinet practice and minimum daily practice of two hours [24]. The presence of beard was also defined as exclusion criterion for possible interference with the skin surface temperature measured by the infrared camera. The musicians selected belonged to an age group ranging from 18 to 49 years of age (mean of 25, 5 years old). The number of years of practice of the instrument covered an interval between 8 and 37 years.

### 2.1. Thermographic Study

For the acquisition of the thermographic images, a document containing a set of indications was given to the participants in advance. In the previous 24 h of the appointment, the use of anti-inflammatories, analgesics, corticosteroids, or any other substance that could alter neuropsympathetic function should be avoided The participants are also instructed that on the day of the examination, should avoid hot drinks (e.g., coffee, tea) for at least 2 h before the thermal images are captured, any jewelry have to be removed, and the application on the face of any type of make-up/creams and perfumes should be avoided, as well. The male participants must be shaved.

**Figure 1:**
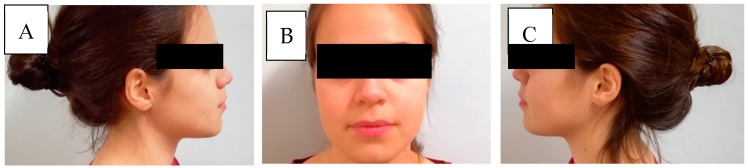
Participant’s extra-oral photographs. (**A**) Front view; (**B**) Right side view; (**C**) Left side view.

In order to aid in the analysis of thermographic images, the capture of extra-oral and intra-oral photographs was included in the data collection process. Extra-oral photographs of the face were taken in frontal, right lateral and left lateral views, using a photographic camera (Figure 1).

**Figure 2:**
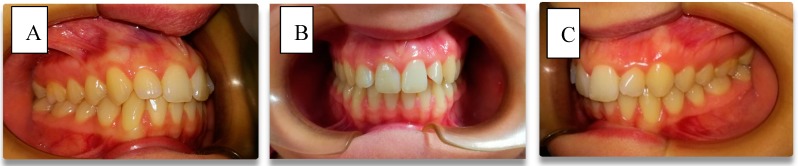
Intraoral photographs of the participant. (**A**) Right side view; (**B**) Front view; (**C**) Left side view.

Subsequently, intra-oral photographs of each patient were performed. The dentoalveolar components were also recorded in frontal, right lateral and left lateral views (Figure 2).

Before the image collection began, the selected musicians remained in the room for an acclimatization period of 15 min to ensure the adaptation of the musician’s body temperature with the environment. The ambient temperature and humidity were recorded during the tests with a thermometer TESTO 175-H1 (Transfer Multisort Elketronik, Lodz, Poland). A special attention was also taken in order to close the door and cover the windows of the room, so that there was no sudden change in temperature neither incident natural light.

**Figure 3:**
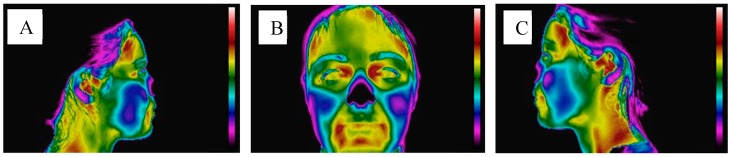
Thermographic images of the resting face. (**A**) Front view; (**B**) Right side view; (**C**) Left side view. It is possible to observe the temperature scale on the right side of each image, where the color black/purple corresponds to the minimum temperature value, while the color red/white corresponds to the highest temperature value.

The acquisition of the thermographic images was done using the Flir^®^ E60sc camera (Flir Systems, Inc., Wilsonville, EUA). The patient was instructed to place the upper lip slightly touching the lower lip, adopting a relaxed position with the teeth in disocclusion. In order to standardize the individuals’ posture during this procedure, the mean sagittal plane was perpendicular to the horizontal plane, and the Frankfurt plane parallel to the horizontal plane, being used as a reference in order to place the head correctly. The distance for this capture was the same for all individuals and about 1 m [25]. For each participant, thermographic images of the cranio-cervico-mandibular complex were obtained in frontal, right lateral and left lateral views (Figure 3).

**Figure 4:**
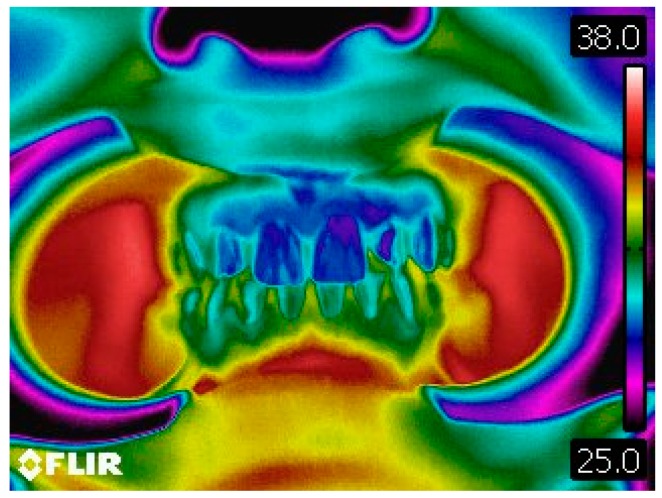
Thermographic image of the anterior dentoalveolar components.

Intraoral thermographic images of the anterior dentoalveolar components were also obtained (Figure 4).

After the acquisition of the extra-oral and intraoral photographs, as well as the thermographic images of the clarinetist at a rest position, he/she start a musical performance of a piece entitled “Vingt Études”, previously selected due to technical requirements. The musical rehearsal implied in this study was capable of generating substantial differences on the muscular groups of the CCMC that can be quantified with the aid of infrared thermography. The time duration of the musical performance was 10 min, which means that the musician had to repeat the performance a second time without any stop.

**Figure 5:**
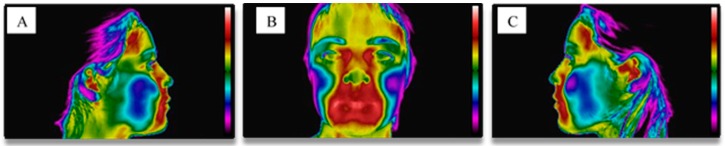
Thermographic images of the face after the musical performance. (**A**) Right side view; (**B**) Front view; (**C**) Left side view. It is possible to observe the temperature scale on the right side of each image, where the color black/purple corresponds to the minimum temperature value, while the color red/white corresponds to the highest temperature value.

**Figure 6:**
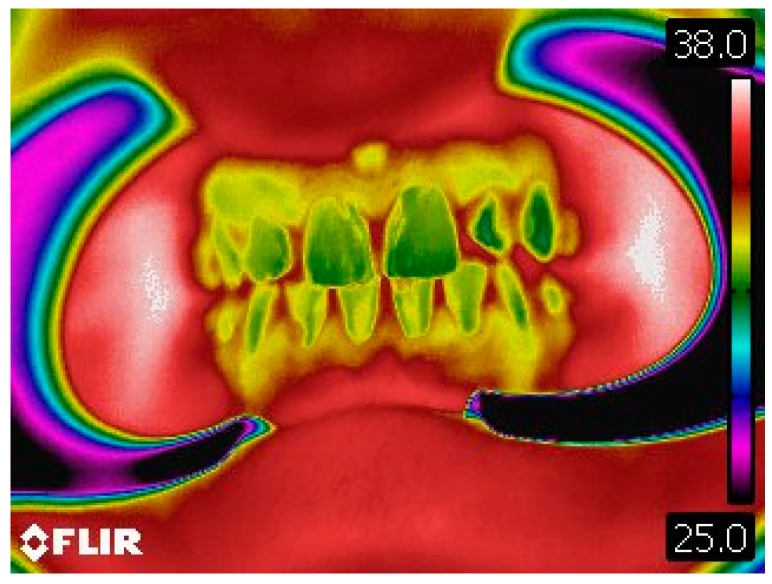
Thermographic image of anterior dentoalveolar components after musical performance.

After the execution of the musical rehearsal, new thermographic images of the frontal, right lateral and left lateral views were taken, as well as the anterior dentoalveolar components view (Figure 5 and Figure 6).

**Figure 7:**
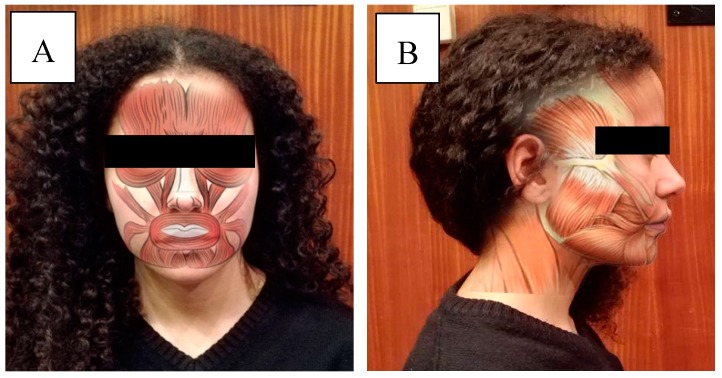
Representation of the anatomical zones of interest. (**A**) Frontal view of the facial muscles; (**B**) Lateral view of the facial and masticatory muscles.

The software Flir^®^ Researcher Pro 2.10 was used to analyze the thermographic images. The anatomical structures and regions of interest analyzed were as follows (Figure 7):

1. Temporal muscle (anterior portion);
2. Muscle of the anterior neck triangle (TAP);
3. Masseter (superficial) muscle;
4. Temporomandibular joint (TMJ);
5. Orbicular muscles of the mouth (labial part and marginal part);
6. Perioral teguments;
7. Dentoalveolar components (upper central incisors);

### 2.2. Statistical Analysis

A statistical analysis was performed using the software package IBM SPSS Statistics 25. In the descriptive analysis, a frequency analysis was performed by number of participants (*N*), mean values, standard deviation, minimum, maximum and 25th percentiles, and 75 for each of the variables to be studied. To evaluate if the variables followed the normal distribution, the Kolmogorov-Smirnov test (K-S) was used. However, given the small sample size (*N* = 30), they were adopted non-parametric methods to verify the effect of the variables under studied. In this way, it was possible to compare independent samples using Wilcoxon and Mann-Whitney tests. For the correlation between the variables, the Spearman correlation coefficient (*ρ*) was used. Decisions were made using a 95% confidence level.

All subjects gave their informed consent for inclusion before they participated in the study. The study was conducted in accordance with the Declaration of Helsinki, and the protocol was approved by the Ethics Committee of Faculty of Dental Medicine of University of Porto (000485).

## 3. Results

### 3.1. Statistical Analysis Concerning the Thermographic Study

In order to verify statistical evidence (*p* < 0.05) between before and after musical performance, rejecting the null hypothesis, the *Wilcoxon* test was applied. Considering the results of this test, it was possible to verify that there is statistical evidence between “before” and “after” musical performance in the left temporal muscle, orbicularis muscle (marginal part), left and right perioral teguments, as well as teeth 1.1 and 2.1. There is also statistical evidence regarding the final and initial asymmetries regarding temporal muscle and orbicular muscles (labial and marginal) (Table 1).

On the other hand, in order to verify the existence of influence of the degree of asymmetry, a convention value was chosen, with a value greater than 0.3 °C and the non-parametric Mann-Whitney H test was applied. Table 2 shows the anatomical zones where there is statistical evidence of rejection of the null hypothesis and indication of influence on the degree of asymmetry for possible changes at the CCMC level.

1. Temporal muscle (anterior portion);
2. Muscle of the anterior neck triangle (TAP);
3. Masseter (superficial) muscle;
4. Temporomandibular joint (TMJ);
5. Orbicular muscles of the mouth (labial part and marginal part);
6. Perioral teguments;
7. Dentoalveolar components (upper central incisors);

To analyze the correlations between the studied variables and the measured thermal variables, Spearman’s correlation coefficient was used, with significance values of 0.05 (weak) and 0.01 (strong). The following Table 2 shows the rho values for the correlations with statistical evidence of significance.

### 3.2. Degree of Asymmetry Present in Regions of Interest to CCMC

**Figure 8:**
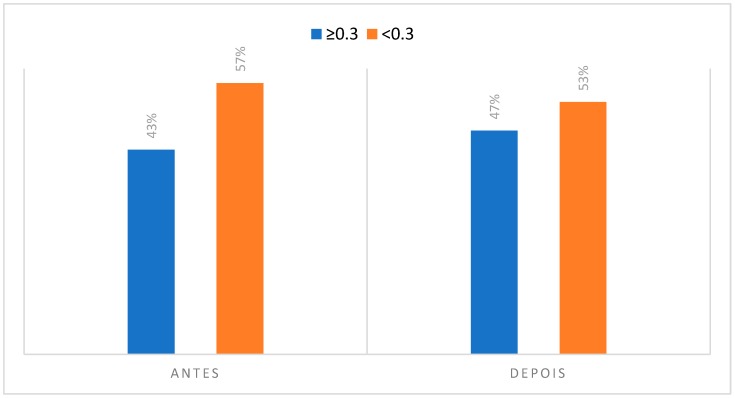
Degree of asymmetry present in the region corresponding to the temporal muscle in the initial phase, at rest position and after the musical performance.

**Figure 9:**
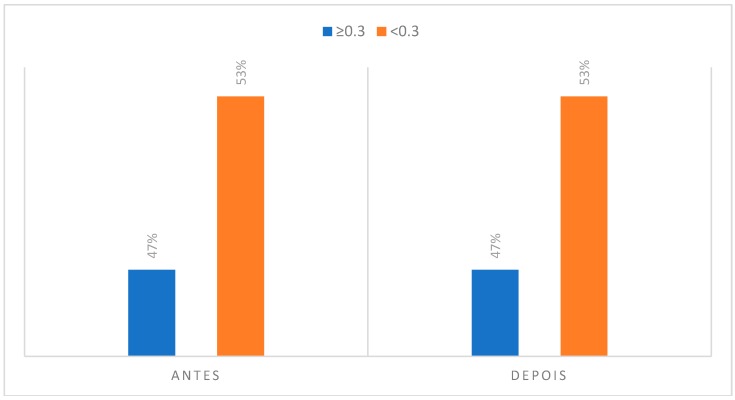
Degree of asymmetry present in the region corresponding to the TAP in the initial phase, at rest position and after the musical performance.

**Figure 10:**
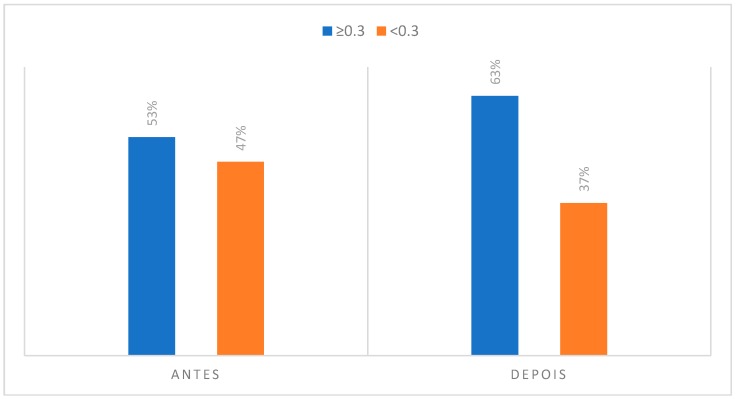
Degree of asymmetry present in the region corresponding to the masseter muscle in the initial phase, at rest position and after the musical performance.

**Figure 11:**
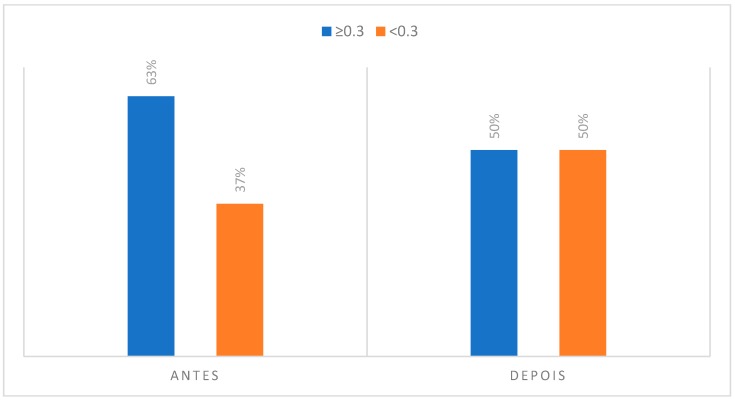
Degree of asymmetry present in the region corresponding to TMJ in the initial phase, at rest position and after the musical performance.

**Figure 12:**
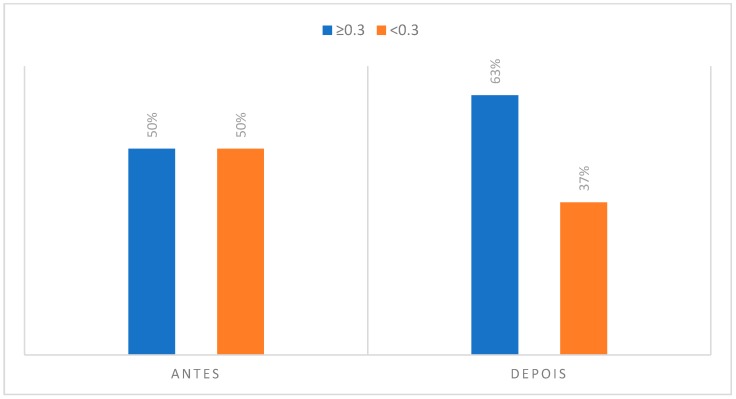
Degree of asymmetry present in the region corresponding to the orbicularis muscles in the initial phase, at rest position and after the musical performance.

**Figure 13:**
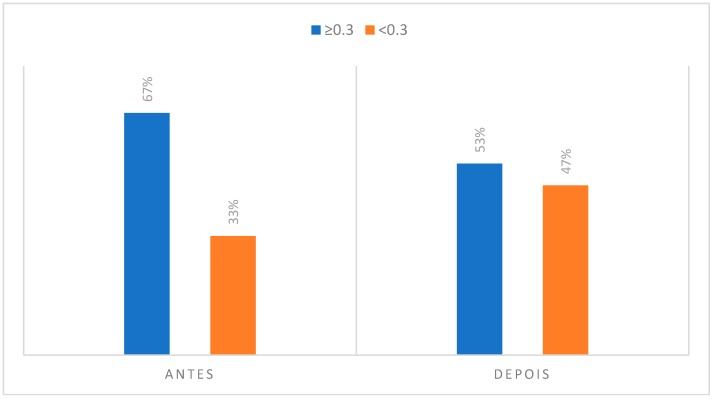
Degree of asymmetry present in the region corresponding to the perioral integuments in the initial phase, at rest position and after the musical performance.

**Figure 14:**
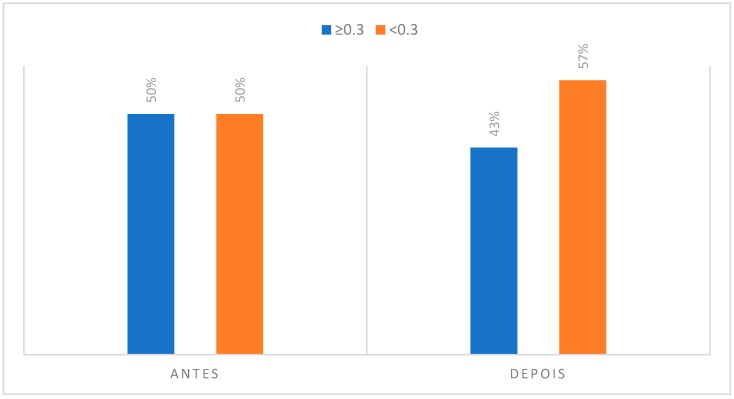
Degree of asymmetry present in the region corresponding to the dentoalveolar components in the initial phase, at the rest position and after the musical performance.

The following Figure 8, Figure 9, Figure 10, Figure 11, Figure 12, Figure 13 and Figure 14 are representative of the degree of asymmetry present in the regions of interest of CCMC in the initial stage of rest and after the individual’s musical performance.

#### (1) Temporal Muscle

#### (2) Anterior Triangle Musculature of the Neck

#### (3) Masseter Muscle

#### (4) Temporomandibular joint (TMJ)

#### (5) Orbicular Muscles (Labial and Marginal Components)

#### (6) Perioral Teguments

#### (7) Dentoalveolar Components (Upper Central Incisors)

## 4. Discussion

This work aimed to clarify questions related to the musical performance of clarinetists and their inherent functional dependence with the stomatognathic apparatus. For this, infrared medical thermography technique was used to quantify the temperature present in the areas corresponding to the different constituent structures of the CCMC.

Actually most of the work related to the health of orchestra instrumentalists has mainly focused on the musculoskeletal apparatus and analyzed psychosomatic issues [16,18,19]. In relation to musculoskeletal disorders, there was a greater predominance of the study of the upper limbs, lumbar region, and cervical zone [7,8]. In fact, the orofacial structures did not receive attention from the medical area and performing arts. As such, most of the wind instrumentalists were unaware of some of the relevant issues associated with their musical activity, where the intervention and role of the dentist may become useful. One of the most significant examples concerns the excessive use of certain muscular groups of the head and neck, such as the masseter muscle, the anterior temporal muscle, and the sternocleidomastoid muscle. It is known that the primary functions of the muscles constituting the stomatognathic apparatus are mastication, swallowing, and phonation, so it is possible to conjecture that a musician who performs his musical practice of clarinet with more than 3 h of daily practice will have a hyperactivity of these muscles. Likewise, the sternocleidomastoid muscle that is associated with neck stabilization during the clarinetist’s musical performance may be in hypertonia to promote the stabilization of the cervical zone, with a continuous contraction.

The adoption of incorrect postures and repetitive movements by clarinetists makes this target population susceptible to possible changes in the CCMC, which in turn, may limit the professional performance of the musician. Thus, the dentist may assume a preponderant role in execution a clinical examination, as well as a detailed clinical history, where the inclusion of these variables should be taken into consideration for the complete study of clarinetist. The use of diagnostic aids, such as thermography, will help clinicians to achieve a favorable interpretation of the anatomy and physiology that the involved regions of interest of the CCMC have on the activity of a clarinet player.

Steinmetz et al. studied the frequency of temporomandibular disorder presence in a population of 408 musicians. The questionnaire delivered shows that the pain associated with musical performance was present in the region of the teeth or jaws in a percentage of 19–47% of the musicians. The presence of pain at the temporomandibular joint level was also reported in 15–34% of the study’s target elements. The authors of this study referred to the importance of promoting alert mechanisms among the instrumentalists of the orchestra to a greater degree of knowledge of the temporomandibular dysfunction, making it possible to act more quickly in their diagnosis and treatment.

On the other hand, Nishiyama et al. studied a group of non-professional wind instrumentalists, where they found that the difference in the degree of prevalence of temporomandibular disorders was not significantly different among the group of instrumentalists (29.2%) of the control group (21.2%). It was also found that the frequency of individuals who reported the pressure exerted by the mouthpiece was included in a high-risk group for the presence of temporomandibular disorder, 47.7%, compared to the other group with a low probability of the presence of these disorders 21.6%. Thus, it was possible to verify that the pressure exerted by the mouthpiece seems to be a contributing factor for the presence of temporomandibular disorders.

Steinmetz et al. demonstrated in another study, based on 36 university musicians, that more than 80% of these musicians had already experienced an episode of pain in relation to the musculoskeletal apparatus. Stechman Neto et al. also addressed the issue of musculoskeletal disorders, especially those of the temporomandibular joint, where, in an analysis of 39 musicians, 42.3% reported having teeth closing their consciousness, 25% reported temporomandibular joint pain, and 42% clicks.

On the other hand, Van Selms et al. have shown that there is limited evidence to conclude that playing a wind instrument could pose a risk to the temporomandibular joint. Grammatopoulos et al. attempted to study the effect of blow instrument practice on the level of occlusion, where they concluded that there is no significant influence on the position of the anterior tooth and that this practice will not be an important etiological factor in the development of a malocclusion.

In view of the above, this research work is aimed to complement the information already available regarding the orofacial structures and wind instrumentalists, but with the premise of allowing the introduction of new technologies that can be useful for performing arts medicine. The introduction of thermography in this area had already been performed previously, proving an effective and determinant method in the analysis of these structures [20,21,22,23].

Thermography provides the measurement of skin temperature representative of the thermal events that occur beneath the surface [24]. In the last five decades, there has been a significant increase in the use of thermal cameras in order to establish possible correlations between physiology and skin temperature. This infrared technique has been used with relevance in the diagnosis of diabetes, neuropathies, and peripheral vascular disorders. It has also been used in the detection of problems related to the medical areas of gynecology, dermatology, and cardiology [2].

Recent studies have demonstrated new applications for this technique, namely monitorization and prevention of sports injuries in modalities such as handball, soccer, and swimming [25,26]. Thermography is accurate and reliable as a complementary diagnostic tool, if one considers the theory of the musculoskeletal system according to which the structures must be in thermal equilibrium when in a healthy state. On the other hand, it is known that myofascial trigger points and temporomandibular disorders cause regional sympathetic hyperactivity at local temperatures due to cutaneous vasomotor activity, so thermography has been applied in these cases to detect functional alterations [27].

In this work, other orofacial structures, to our knowledge, had not yet been referenced and analyzed to date, as was the case of dentoalveolar components. This situation arose as a result of the pressures of the clarinet mouthpiece inside the mouth during the embouchure.

Thus, the analysis of the thermograms allowed to verify that there were statistically significant differences in the moment before and the moment that followed the instrumental practice, namely in the left temporal muscle, in the orbicularis muscle (labial part), in the left and right perioral integuments, as well as on teeth 11 and 21. There was also statistical evidence regarding the final and initial asymmetries regarding temporal muscle and orbicular muscles (labial and marginal).

Considering the temporal muscle is a retrusive elevator muscle, responsible for the fine movements of the mandible, we could verify that during the musical performance this muscle is working to attain different pitches, being able to present differences as far as the asymmetries of temperature before and after the musical performance. This area of interest presented 43% of the individuals with an asymmetry greater than, or equal to 0.3 °C, in the initial phase, increasing to 47%, after the instrumental practice.

In the case of the temporomandibular joint, there was a decrease in the value of individuals with more than 0.3 °C of asymmetry, from 63% to 50%, something that may in fact be associated with the TMJ biomechanics during the embouchure. In this situation, an initial rotation of the condyle-disc complex may occur, followed by a translation that allows the mouth to open while the mouthpiece rests on the palatal aspect of the upper incisors. In case the individuals do not present any type of TMJ pathology, there may be an increase in the symmetrical temperature in relation to the movement of the condyle in the glenoid cavity. It is assumed that the excursion movement is as symmetrical as possible between the two condyles.

Concerning the masseter muscle, the thermographic study evidenced that in the initial phase of rest, it presented an asymmetry of 53% among clarinetists. This muscle group may, in turn, perform an isometric contraction during the stabilization of the mouthpiece in the oral cavity, something that may not be equivalent in terms of pressures and forces exerted causes an increase to 63% of individuals with an asymmetry of more than 0.3 °C. There have been previous studies that describe the usefulness of the application of infrared thermography in the diagnosis and evaluation of the treatment plan of a temporomandibular disorder in a clarinet player [28].

The anterior triangle of the neck is a muscular area that is stable during the instrumental practice, since during the performance there are no large oscillations in the cervical position of the clarinetist, namely some type of rotation of the neck. Usually, the clarinet player holds the instrument with a slight elevation of the left shoulder relative to the right shoulder, but the head remains stabilized looking at the score and there is no significant rotation of the cervical region in function of the instrument. Such a situation may be associated with the fact that the anterior neck triangle presents 43% of the individuals with an asymmetry greater than 0.3 °C before the musical performance, maintaining this same value after the performance.

Relative to the analysis of the thermograms related to the area of interest of the orbicularis muscles, it was verified that 50% of the subjects studied had an asymmetry greater or equal to 0.3 °C before their musical activity. However, this value increased to 63% of individuals after performing the musical piece. In view of the above, such an event may be justified by the pressures exerted during the labial sealing that occurs. In addition, according to the participants, the lower lip is the anatomical zone most referenced for the presence of pain with 97% of the cases recorded. It was also possible to note that 73% of the participants reported some type of discomfort on the lower lip.

Contrary to all other regions of interest in the thermographic study, the perioral teguments and dentoalveolar components suffered a decrease in the number of individuals who presented an asymmetry greater than or equal to 0.3 °C. That is, in the case of the perioral teguments before musical performance there was a percentage of 67% of individuals with a temperature differential of more than 0.3 °C and, after musical performance this value decreased to 53%. Regarding the analysis of the dentoalveolar components, namely at the upper incisor level, there was also a decrease in the number of individuals with a temperature asymmetry greater than or equal to 0.3 °C when compared to the initial moment of acquisition of the infrared images with the final moment. It can also be noted that 77% of clarinetists reported pain and/or discomfort in relation to the dentoalveolar components, 66.7% of which were in teeth 21 and 11.

The explanation of the phenomenon occurring in these last two areas of interest may be related to the decrease in blood flow that occurs when a given force is applied. According to the literature and considering a light force, the pressure exerted by the mouthpiece towards the dentoalveolar components and teguments, the applied force may be the origin of a decrease in blood flow, displacement of periodontal ligament fluids, as well as displacement of the tooth into the alveolus. Such events occur during the initial seconds after the application of a light force [29,30].

Therefore, the thermographic study of the orofacial structures involved in clarinetists musical performance, with a sample of 30 clarinetists, can bring valid information concerning the interrelationship of the wind instrumentalists’ mouthpiece, the embouchure and the anatomical regions of interest of the CCMC. The musical gesture of the musician and the interface of such areas like the dentoalveolar components are to our knowledge and till this moment an innovative approach in order to quantify substantial differences that can be present before and after playing a wind instrument. The fact that this experimental design also included the thermographic analysis after a dynamic test like the musical activity is in the authors’ opinion an added value to the dentistry area, since we can observe and quantify what is really happening on vital areas for the wind instrumentalist’s embouchure. Measuring these occurrences will provide a better understanding of the clarinetists’ embouchure and enable dentists to be better prepare to treat this highly demanding group of professionals.

## 5. Conclusions

The CCMC thermographic standards involved in the musical practice of clarinetists can serve as a predictive analysis of the susceptibility that each musician will have in relation to regions in overuse.

The analysis of the thermograms allowed us to conclude that there are statistically significant differences in the areas corresponding to the left temporal muscle, the orbicularis muscle (labialFoto part), the left and right perioral teguments, as well as in the teeth 11 and 21.

There is also statistical evidence regarding the presence of asymmetries ≥0.3 °C on the initial and final thermograms regarding temporal muscle and orbicular muscles (labial and marginal).

## Figures and Tables

**Table 1:** Influence of the degree of asymmetry in the regions of interest with statistical significance (*p* < 0.05).

Region of Interest	*p* Value	
	Before	After
1. Temporal muscle	0.035	0.016
2. Muscle of the anterior neck triangle	0.022	0.004
3. Masseter (superficial) muscle	0.010	
4. Temporomandibular joint (TMJ)		0.024
5. Orbicular muscles of the mouth	0.005	
6. Perioral teguments		0.007
7. Dentoalveolar components	0.012	0.007

**Table 2:** Correlation of the hours of practice with the thermal variables.

Thermal Variables	Spearman Correlation (*ρ*)	
	Before	After
Right temporal muscle	−0.407 *	
Right temporomandibular joint	−0.363 *	
Left perioral teguments	−0.417 *	−0.418 *
Tooth 11	−0.399 *	−0.423 *
Tooth 21	−0.465 **	−0.396 *
Orbicular muscle—marginal component		−0.383 *

* *p* < 0.05; ** *p* < 0.01.
